# Modifications of Resorbable Root Canal Filling Materials for Primary Teeth: A Systematic Review

**DOI:** 10.3390/ma19050950

**Published:** 2026-02-28

**Authors:** Anna Błaszczyk-Pośpiech, Sylwia Kiryk, Natalia Nawrot, Julia Kensy, Jan Kiryk, Agnieszka Kotela, Magdalena Wawrzyńska, Maria Szymonowicz, Jacek Matys, Maciej Dobrzyński

**Affiliations:** 1 Pre-Clinical Research Center, Wrocław Medical University, Karola Marcinkowskiego 1, 50-368 Wrocław, Poland; magdalena.wawrzynska@umw.edu.pl (M.W.); maria.szymonowicz@umw.edu.pl (M.S.); 2 Department of Pediatric Dentistry and Preclinical Dentistry, Wrocław Medical University, Krakowska 26, 50-425 Wrocław, Poland; s.roguzinska@gmail.com (S.K.); julia.kensy@student.umw.edu.pl (J.K.); maciej.dobrzynski@umw.edu.pl (M.D.); 3 Medical Center of Innovation, Wrocław Medical University, Krakowska 26, 50-425 Wroclaw, Poland; nawrotnat@gmail.com (N.N.); kotela.agnieszka@gmail.com (A.K.); 4 Dental Surgery Department, Wrocław Medical University, Krakowska 26, 50-425 Wrocław, Poland; jan.kiryk@umw.edu.pl

**Keywords:** primary teeth, root canal filling material, modifications, pulpectomy, antimicrobial agents

## Abstract

**Objective:** This systematic review aimed to evaluate material-based modifications of resorbable root canal filling materials for primary teeth, assessing how compositional changes—including bioactive additives, antimicrobial agents, and alternative base matrices—influence antimicrobial performance. **Methods**: A systematic search of PubMed, Scopus, Web of Science (WoS), and Embase was performed in October 2025. Search terms included (primary teeth OR deciduous teeth) AND (root canal filling materials OR root canal filling OR canal obturation) AND (antibacterial agents OR antibacterial OR antimicrobial). Study selection adhered to PRISMA 2020 standards and was systematically organized through the PICO framework. From 199 identified records, 18 studies met the eligibility criteria. **Results**: Most studies evaluated modified zinc oxide-based materials. Additives such as propolis, *Morinda citrifolia* extract, *Aloe vera*, and olive oil enhanced antimicrobial activity or improved clinical and radiographic outcomes compared with conventional zinc oxide–eugenol. Triclosan-containing formulations consistently demonstrated strong antibacterial effects. In contrast, chlorhexidine yielded variable results, with some calcium hydroxide–based pastes showing superior performance in its absence. Antibiotic-enriched materials exhibited high antimicrobial efficacy; however, several studies raised concerns regarding the potential development of bacterial resistance. **Conclusions**: Most of the introduced modifications of resorbable root canal filling materials for primary teeth enhance antimicrobial activity and their physicochemical properties in vitro. Clinical evidence is limited and heterogeneous, and therefore, its superiority over conventional materials cannot be definitively determined. Further long-term, randomized clinical trials on large patient groups, evaluating the same modifications, are needed to confirm the effects observed in laboratory studies.

## 1. Introduction

The maintenance of deciduous teeth until their physiological exfoliation is a fundamental objective in pediatric dentistry. Premature loss of primary teeth may lead to a range of adverse outcomes, including disturbances in the development and eruption of permanent successors, impaired speech development, compromised masticatory function, loss of arch space, occlusal discrepancies, ectopic tooth eruption, altered tongue habits, and unfavorable esthetic consequences [1,2,3]. When feasible, endodontic treatment of primary teeth affected by necrosis or irreversible pulpitis is considered the most effective method to prevent these sequelae and maintain normal oral function [4,5]. Despite its benefits, endodontic management in primary teeth presents unique challenges. The complex anatomy of their root canal system—characterized by apical deltas, lateral canals, and numerous anastomoses—together with ongoing physiological root resorption and the frequent difficulty in obtaining adequate cooperation from young patients, makes treatment technically demanding [5,6,7,8]. Nevertheless, successful pulpectomy permits the maintenance of primary teeth until their natural exfoliation in most cases, thereby promoting normal occlusal development and the proper eruption of permanent teeth [9].

Pulpectomy involves the complete removal of both coronal and radicular pulp tissue and is indicated in cases of irreversible pulpitis or pulp necrosis in primary teeth exhibiting minimal or no physiological root resorption [10,11,12]. Contraindications include the presence of cystic lesions, extensive crown destruction, pulpal floor perforations, and certain systemic conditions—particularly immunodeficiencies and congenital heart defects [13,14]. After pulp extirpation, the canals must be carefully instrumented using stainless-steel hand files or rotary nickel-titanum (NiTi) instruments, with special consideration for the developing permanent tooth germ situated near the apices of primary roots [15,16]. Mechanical preparation is complemented by irrigation with commonly used solutions such as 2% chlorhexidine (CHX), 1–2.5% sodium hypochlorite (NaOCl), 17% ethylenediaminetetraacetic acid (EDTA), or 6% citric acid [17,18,19]. The final step of pulpectomy is obturation of the prepared canals. The ideal obturation material for primary teeth should be biocompatible, resorb at a rate comparable to natural root resorption, remain dimensionally stable, be harmless to the permanent successor, adhere adequately to canal walls, exhibit antibacterial and antiseptic properties, be easily retrievable, when necessary, radiopaque, and not cause discoloration. At present, no available material fulfills all of these requirements [20,21]

**Figure 1:**
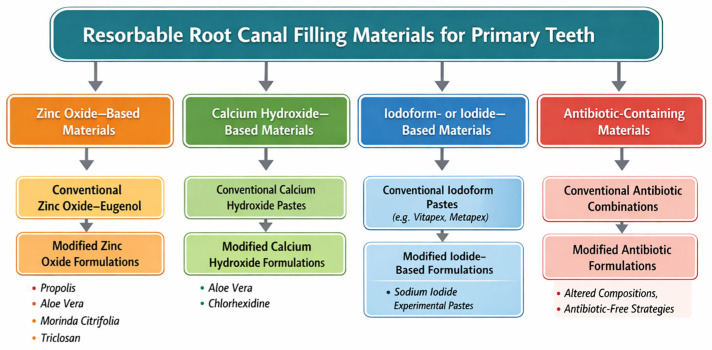
Classification of root canal filling materials for primary teeth, including conventional materials and recently developed multi-component or modified formulations.

Currently, a variety of resorbable root canal filling materials are used in the treatment of primary teeth, including zinc oxide–eugenol, iodoform-based pastes, calcium hydroxide formulations, and ready-to-use multi-component materials such as Endoflas [2]. Antibiotic-containing pastes [1,5] and more recently developed bioceramic-based materials, such as Bio-C Pulpecto, have also been introduced [22]. Although each of these materials has demonstrated clinical success, all present notable limitations. Some resorb either too rapidly or too slowly relative to physiological root resorption, which may compromise treatment outcomes [23,24,25]. Zinc oxide–eugenol, for example, resorbs more slowly than many other materials [23,26] and its eugenol component has been reported to exert cytotoxic effects on fibroblasts and osteoblast-like cells, potentially impairing tissue healing [24,27]. Iodoform-containing pastes may cause crown discoloration [15] and have been associated with cellular stress responses [28]. Calcium hydroxide, while widely used, can alter dentin structure and decrease root strength during prolonged contact [29,30,31,32], and it often resorbs faster than the primary root itself [6]. Other formulations have shown limited antibacterial activity and suboptimal biocompatibility, reducing their predictability in clinical use. For these reasons, recent research has focused on modifying existing materials to overcome such shortcomings [5,33]. Proposed enhancements include the incorporation of natural bioactive additives such as propolis [24,34,35] and *Aloe vera* [4,25,36], as well as antibiotics [1] and antiseptic agents [2,37,38]. Overall, these modifications aim to optimize physicochemical properties, improve biocompatibility, enhance antimicrobial effectiveness, and achieve more favorable in vitro and in vivo clinical outcomes while minimizing adverse tissue reactions. The classification of resorbable root canal filling materials used for primary teeth was presented in Figure 1.

The objective of this systematic review was to evaluate modifications of resorbable root canal filling materials for primary teeth. In vitro and in vivo studies were analyzed independently, as they represent fundamentally different methodological designs and have distinct clinical relevance. In vitro studies were analyzed to assess antimicrobial and physicochemical properties, whereas in vivo investigations were evaluated separately to determine clinical and radiographic outcomes following pulpectomy. Recent systematic reviews have evaluated the clinical effectiveness of root canal filling materials used in primary teeth, focusing primarily on overall treatment success rates and comparisons between commercially available formulations [39,40]. However, these reviews did not specifically address the role of material modifications, experimental additives, or compositional optimization strategies aimed at enhancing antimicrobial properties and biological performance. In contrast, the present systematic review uniquely focuses on modifications of resorbable root canal filling materials, including both experimental and clinically applied formulations. By systematically analyzing in vitro studies that assess antimicrobial and physicochemical properties alongside in vivo clinical studies evaluating radiographic and clinical outcomes, this review provides an integrated synthesis of laboratory and clinical evidence. This approach allows for the identification of translational gaps and highlights how specific material modifications may influence clinical performance in primary teeth.

## 2. Materials and Methods

### 2.1. Focused Question

This systematic review was structured according to the PICO framework [41]. The focused question was: In primary teeth or experimental modes relevant to pediatric endodontics (Population), do modified resorbable root canal filling materials (Intervention) demonstrate improved clinical, radiographic, or laboratory-based biological and physicochemical outcomes (Outcome) compared with conventional, unmodified resorbable root canal filling materials (Comparison)?

### 2.2. Protocol

**Figure 2:**
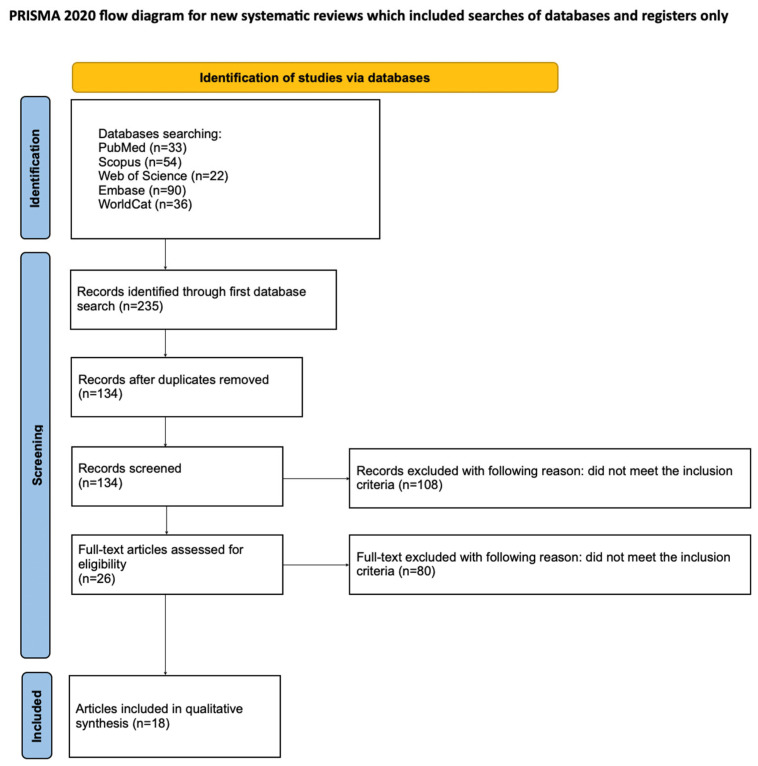
PRISMA flow diagram [42].

The article selection process for the systematic review was systematically detailed using the PRISMA flow diagram (Figure 2). The systematic review was registered with the Open Science Framework under the following link: https://osf.io/gn5fw (accessed on 19 November 2025).

### 2.3. Eligibility Criteria

The review included studies that satisfied the following criteria [43,44,45,46,47]:

- Studies evaluating resorbable root canal filling materials in primary teeth;
- Studies investigating modifications to root canal filling materials (e.g., addition of antibacterial agents, bioactive materials, nanoparticles, or other additives);
- In vitro experimental studies;
- In vivo including non-randomized controlled clinical trials (NRSs) and Randomized controlled clinical trials (RCTs);
- Studies including a control group, such as conventional unmodified resorbable root canal filling materials.
- Studies published in English.

The reviewers established the following exclusion criteria [43,44,45,46,47]:

- Studies focusing on endodontic treatment of permanent teeth;
- Studies evaluating non-resorbable root canal filling materials (e.g., gutta-percha, resin-based sealers intended for permanent teeth);
- Case reports, clinical reports, editorials, letters or opinion papers;
- Narrative reviews, systematic reviews or meta-analyses;
- Studies without accessible full-text;
- Duplicated publications

### 2.4. Information Sources, Search Strategy, and Study Selection

A comprehensive electronic search was conducted in October 2025 in four bibliographic databases (PubMed, Scopus, WoS, and Embase) and supplemented by a gray literature search in WorldCat. The search strategy was designed to identify studies evaluating modifications of resorbable root canal filling materials used in primary teeth. Search terms were applied to titles, abstracts, and controlled vocabulary/subject headings (when available), combining concepts related to primary dentition, root canal filling/obturation materials, and antimicrobial properties.

The search strategy used for each database was as follows:

PubMed: (“primary teeth” [MeSH Terms] OR “deciduous teeth” [MeSH Terms] OR “primary teeth” [Title/Abstract] OR “deciduous teeth” [Title/Abstract]) AND (“root canal filling materials” [MeSH Terms] OR “root canal filling” [Title/Abstract] OR “canal obturation” [Title/Abstract]) AND (“antibacterial agents” [MeSH Terms] OR “antibacterial” [Title/Abstract] OR “antimicrobial” [Title/Abstract]).

Scopus: (TITLE-ABS-KEY (“primary teeth” OR “deciduous teeth”)) AND (TITLE-ABS-KEY (“root canal filling” OR “canal obturation”)) AND (TITLE-ABS-KEY (“antibacterial” OR “antimicrobial”)).

WoS: (TS = (“primary teeth” OR “deciduous teeth”)) AND (TS = (“root canal filling” OR “canal obturation”)) AND (TS = (“antibacterial” OR “antimicrobial”)).

Embase: (‘primary tooth’/exp OR ‘deciduous tooth’/exp OR “primary teeth”:ab,ti OR “deciduous teeth”:ab,ti) AND (‘root canal filling’/exp OR “root canal filling”:ab,ti OR “canal obturation”:ab,ti) AND (‘antibacterial agent’/exp OR “antibacterial”:ab,ti OR “antimicrobial”:ab,ti).

WorldCat: (primary teeth OR deciduous teeth) AND (root canal filling OR canal obturation) AND (antibacterial OR antimicrobial).

All retrieved records were imported into a Microsoft Excel (version: 15.0.4569.1504, Microsoft Corporation, Redmond, WA, USA). spreadsheet, where duplicate entries were removed. The remaining studies were screened by title and abstract according to predefined eligibility criteria. Articles that appeared eligible were subsequently evaluated in full text. Only studies with available full-text versions that met the inclusion criteria were included in the final qualitative synthesis.

Although antimicrobial-related terms were included in the search strategy, they were intentionally used to increase sensitivity for identifying studies on modified resorbable root canal filling/obturation materials used in primary teeth, as antimicrobial enhancement is the most commonly reported rationale for such modifications in pediatric endodontics. Importantly, data extraction was not restricted to antimicrobial outcomes: physicochemical properties, biocompatibility, resorption characteristics, and clinical or radiographic outcomes were systematically extracted and analyzed at the full-text stage whenever reported, regardless of whether these terms were explicitly included in the search strategy.

### 2.5. Data Collection Process and Data Items

Six independent reviewers (A.B.-P., J.K., A.K., S.K., J. Kensy., and N.N.) screened the studies individually to determine which publications fulfilled the established inclusion criteria. For every article deemed eligible, relevant data were collected on the first author, year of publication, study design, study title, characteristics of the modified resorbable filling materials. Data extraction included all reported outcomes, including physicochemical, biological, clinical, and radiographic parameters, regardless of the terminology used in the original search strategy. All collected information was then systematically organized and documented using a standardized Excel spreadsheet to ensure consistent data handling and facilitate subsequent analysis. Agreement among reviewers was quantified using Cohen’s kappa statistic. Any discrepancies regarding study inclusion or exclusion were subsequently discussed collectively, and consensus was reached through group deliberation.

### 2.6. Quality Assessment and Risk of Bias

Two blinded reviewers (J.M. and M.D.) independently assessed the methodological quality and a potential risk of bias of all included studies using the Mixed Methods Appraisal Tool (MMAT), version 2018. This validated tool enables standardized evaluation across diverse study designs. Given the inclusion of both in vivo and in vitro studies, MMAT criteria were applied selectively according to study type.

For in vivo studies, all MMAT criteria were applied in full. For in vitro studies, only criteria conceptually applicable to laboratory-based research were assessed, while items related to sample representativeness and nonresponse bias were considered not applicable (N/A) and were therefore not evaluated. This adapted approach was used to provide a structured and transparent appraisal of methodological quality; it should not be interpreted as a definitive risk-of-bias judgment for laboratory-based studies.

Each criterion was scored as “yes,” “no,” or “not applicable”. Disagreements between reviewers were resolved through discussion until a consensus was reached. Inter-rater reliability was evaluated using Cohen’s kappa coefficient, calculated with MedCalc software (version 23.1.7; MedCalc Software Ltd., Brussels, Belgium). The obtained kappa value of 0.91 (*p* < 0.001) demonstrated a high degree of agreement, indicating near-perfect concordance between reviewers.

### 2.7. Data Synthesis and Statistical Analysis

Due to substantial methodological and clinical heterogeneity among the included studies, particularly with respect to study design, types of material modifications, outcome measures, and assessment methods a quantitative meta-analysis was not feasible. Therefore, the results were synthesized using a qualitative narrative approach. To enhance clarity and methodological coherence, findings from in vitro and in vivo studies were analyzed and presented separately.

## 3. Results

### 3.1. Study Selection

The initial search across PubMed, Scopus, Embase, and WoS yielded 199 potentially relevant records. After removing duplicates, 122 unique articles were screened by title and abstract. Of the 27 studies selected for full-text assessment, one did not report study outcomes, one examined only commercially available materials without assessing any modifications, five were study registrations, and two were excluded due to the lack of an accessible full-text English version. Ultimately, 18 studies met the eligibility criteria and were incorporated in the qualitative synthesis. Due to substantial methodological and clinical heterogeneity among the included studies, a meta-analysis could not be performed.

### 3.2. General Characteristics of the Included Studies

The studies analyzed in this systematic review demonstrated substantial heterogeneity in the types and approaches of material-based modifications to root canal filling materials for primary teeth. Out of the total number of analyzed publications (*n* = 18), only one introduced a completely novel obturation material based on sodium iodide [48]. In contrast, the remaining 17 studies focused on modifying the composition of existing materials or commercially available obturation pastes [1,2,4,5,24,25,26,27,34,36,37,38,49,50,51,52,53].

Zinc oxide–based formulations were the most frequently modified materials, reported in 7 studies [4,25,26,27,34,38,49]. This was followed by antibiotic-containing pastes, which were evaluated in 4 studies [1,2,5,50], and calcium hydroxide–based materials, reported in 3 studies [36,51,52]. Additionally, naturally derived additives were used in nine publications, including propolis [24,26,34], plant oils such as olive oil, ozonated olive oil, or eucalyptus oil [27,49], and herbal extracts including *Aloe vera* and *Morinda citrifolia* [4,25,36,38].

Regarding the type of study, ten publications were conducted in vitro [2,4,5,36,37,38,48,49,50,52], while eight involved in vivo clinical evaluation [1,24,25,26,27,34,51,53]. The in vitro experiments primarily focused on antimicrobial or antifungal activity. Candida albicans was assessed in four cases [4,10], Streptococcus mutans in four [2,5,49,52] and *Enterococcus faecalis* in six [2,5,36,37,38,52].

In vivo studies consistently assessed clinical success through the evaluation of clinical outcomes. Spontaneous pain was evaluated in seven publications [1,24,25,26,27,34,53], gingival swelling in four [24,25,27,53], pain on percussion in six [1,24,25,27,34,53] and abnormal tooth mobility in six [1,24,25,26,27,34]. Additional outcomes included the presence of a sinus tract in three publications [24,25,34] and intraoral or extraoral abscesses in two [1,34].

Radiographic analysis was performed in all in vivo studies. The most commonly evaluated radiographic outcomes included changes in interradicular and/or periapical radiolucency, as well as alterations in bone density or periodontal ligament width, when applicable (Table 1).

### 3.3. Main Study Outcomes

To summarize the findings Table 2 was prepared.

#### 3.3.1. In Vitro Studies—Detailed Outcomes

The included in vitro studies primarily focused on evaluating the antimicrobial activity of root canal filling materials [2,4,5,36,37,38,48,49,50,52]. Analyses were performed using both standard reference strains [2,4,37,38,48,52] and microorganisms directly isolated from infected primary root canals [5,36,49,50]. The most frequently assessed pathogens across the studies were *Enterococcus faecalis*, *Streptococcus mutans*, *Staphylococcus aureus*, *Pseudomonas aeruginosa*, and *Candida albicans* [4,5,36,37,49,52].

Among commercial materials, zinc oxide–eugenol (ZOE) cement was the most extensively investigated [4,36,37,49,52]. It demonstrated high and relatively stable antimicrobial efficacy. Several studies reported broad-spectrum antibacterial and antifungal activity of ZOE, particularly against *Staphylococcus aureus*, *Pseudomonas aeruginosa*, and *Candida albicans* [4,49,52]. Compared with calcium hydroxide-based materials and selected iodoform-containing pastes, such as Metapex or Vitapex, ZOE more consistently produced larger inhibition zones and a more predictable antimicrobial effect [36,49,52].

Calcium hydroxide-based materials generally showed limited antimicrobial effectiveness, particularly against *E. faecalis* and certain Gram-negative bacteria [36,52]. The addition of supplementary components (e.g., sodium fluoride or plant extracts) improved their activity, although they usually did not reach the levels observed for ZOE or antibiotic-containing pastes [36].

Modifications of material composition significantly influenced their biological activity. The addition of plant extracts (e.g., *Aloe vera*, *Morinda citrifolia*, neem, eucalyptus oil) generally enhanced effectiveness against selected pathogens [4,36,49], although the effect was strain-dependent. In particular, improved activity was observed against *Pseudomonas aeruginosa* [4], while for *Candida albicans*, some plant-based combinations were less effective than conventional ZOE [4,49]. A similar trend was noted for modifications with compounds of documented antibacterial activity, such as triclosan or chlorhexidine, which significantly increased inhibition zones, especially against *Enterococcus faecalis* [2,37]. This effect gradually decreased over time, although modified materials retained measurable activity for several weeks [37].

Pastes containing antibiotic combinations (e.g., metronidazole, ciprofloxacin, minocycline, or CTZ) generally exhibited stronger antimicrobial activity than reference materials [5,50]. However, their efficacy was clearly dependent on the resistance profiles of the tested strains. In some cases, reduced susceptibility of specific *Streptococcus mutans* or *Enterococcus faecalis* strains was observed, highlighting the importance of local resistance patterns when interpreting results [5]. Moreover, some microorganisms, including *Candida albicans* and *Clostridium ramosum*, were resistant to both antibiotic-modified formulations and selected iodoform-containing pastes [50].

Some studies also evaluated the physicochemical properties of the materials [38,48], revealing significant differences in solubility, radiopacity, viscosity, and injectability. Certain experimental formulations (e.g., containing terpineol) exhibited high solubility and complete dissolution in a short period, potentially limiting their clinical applicability [38]. In contrast, modifications of sodium-iodide-based with lanolin reduced solubility and allowed compliance with ISO standards while maintaining adequate canal-filling properties [48].

**Table 2:** Summary of antimicrobial, clinical, and radiographic outcomes of modified resorbable root canal filling materials included in the systematic review.

Study	Samples/Specimens	Filling Materials	Antibacterial/Antifungal Findings	Radiographic Outcomes	Other Findings
Alashbal et al. [49]	-In vitro, -Primary teeth, -Samples taken from 21 children aged 3–8.	-Zinc oxide + eucalyptus -Zinc oxide eugenol -Metapex	Inhibiting zone in millimeters: *Streptococcus* spp. Zinc oxide eucalyptus 16.4 ± 2.366 Zinc oxide eugenol 17.300 ± 3.743 Metapex 8.000 ± 4.967 *Candida albicans* Zinc oxide eucalyptus 31.200 ± 3.490 Zinc oxide eugenol 30.200 ± 3.259 Metapex 7.400 ± 6.899	No data	No data
Park et al. [48]	-In vitro -Samples of sodium iodide-based pastes after modifications	L0: sodium iodide, calcium hydroxide, silicone oil L5: sodium iodide, calcium hydroxide, silicone oil, lanolin Control-Vitapex	Log CFU/mL L0: ~6.6 L5: ~6.75 Vitapex: ~6.9 Vitapex > L5 > L0 Antimicrobial reduction (%) L0: ~35% L5: ~20% Vitapex: ~5% L0 > L5 > Vitapex	No data	Physicochemical tests: Solubility: L0 > L5 > Vitapex Radiopacity: Vitapex > L5 > L0 Injectability: Vitapex > L5 > L0 Complex Viscosity Vitapex > L5 > L0 pH: -all pH values were approximately 12 Evaluation of root canal obturation: -all materials exhibited comparable ability to fill the root canal up to the apex.
EL-Desouky et al. [27]	-In vivo -90 secondary primary second molars from 108 children aged 4–8. -Teeth are divided into 3 groups of 30 teeth each.	Group I—zinc oxide ozonated olive oil Group II—zinc oxide with olive oil Group III (control)—zinc oxide with eugenol.	No data	Group I: -the most favorable radiographic outcomes, with the highest bone density at 12 months (114.78 ± 29.05) -the greatest reduction in periodontal ligament width (final value 0.17 ± 0.02). Group II: -faster early bone density improvement, with values at 3 months (62.32 ± 11.09) and 6 months (79.43 ± 18.05), -comparable periodontal ligament width at 12 months (0.17 ± 0.02), though the final bone density was lower (106.64 ± 26.20). Group III: -the least favorable outcomes, lower bone density at all time points, reaching 88.86 ± 31.33 at 12 months, -a smaller reduction in periodontal ligament width (0.18 ± 0.03).	Group I: -comparable results with Group II, -a stable success rate of 92.6% and low symptom rates at 12 months. Group II: -the best clinical outcomes, -the highest success at 6 and 12 months (96.4%) and the lowest incidence of symptoms at 12 months. Group III: -the least favorable outcomes, -higher symptom prevalence at 12 months and lower success rates at 6 and 12 months (85.7%).
Pinky et al. [1]	-In vivo, -40 primary teeth from 28 children aged 4–10. -Divided into 2 groups of 20 teeth each.	Group A—antibacterial paste: ciprofloxacin, metronidazole, minocycline, propylene glycol; Group B—antibacterial paste: ciprofloxacin, ornidazole, minocycline, propylene glycol;	No data	Interradicular radiolucency: -progressive improvement in both groups, with greater reduction in Group B at 6 months (30% vs. 35%) and 12 months (60% vs. 55%). Increased/no increased interradicular radiolucency: -at 6 months, no increase was observed in either group. -at 12 months, increased radiolucency occurred only in Group A (10%), while Group B showed no increase (0%).	Pain: -at 3 and 6 months, no pain was reported. -at 12 months, Group A: 10%, Group B: 0%. Intraoral abscess: -initially Group A: 75%, Group B: 70%; -resolved completely by 3 months and remained absent at 6 and 12 months in both groups. Extraoral abscess: -initially Group A: 30%, Group B: 40%; -absent at 3, 6, and 12 months in both groups. Mobility: -initially Group A: 60%, Group B: 65%; -resolved in both groups at 3, 6, and 12 months. Tenderness: -no tenderness at 3 and 6 months -minimal at 12 months in Group A: 10%
Singh et al. [24]	-In vivo, -60 primary molars from 50 children aged 4–9. -Divided into 3 groups of 20 teeth each.	Group 1: Zinc oxide + eugenol (ZOE), Group 2: Zinc oxide + propolis Group 3: Endoflas	No data	Furcation radiolucency: -gradual decrease in unchanged cases, with higher rates of reduction in Groups 2 and 3 at 6 months (45–50% vs. 35% in Group 1) and 9 months (45–55% vs. 35% in Group 1). Furcation radiolucency: -elevated radiolucency observed in Group 1 at 6 and 9 months (20%); Groups 2 and 3 showed minimal elevation (0–5%). -reduction in radiolucency was highest in Group 2 at 9 months (55%), with Groups 1 and 3 showing 45–50%.	Pain: -high pain incidence in 1st week after treatment in all groups -resolved completely by 3 months. Mobility: -minimal mobility was observed 1 week after treatment in Group 1: 5% and Group 3: 5% -at 6 months Group 1: 5% -at 9 and 12 months no mobility in all groups. Sinus manifestation: -initially present in all groups -mostly resolved by 3 months (Group 1: 5%, Group 2: 10%, Group 3: 0%), -absent in all groups at 6 and 9 months, except Group 1: 15% at 6 months. Tenderness on percussion: -1 week after treatment (Group 1: 45%, Group 2: 70%, Group 3: 55%), -resolved completely by 3, 6, and 9 months. Resorption of material: -no resorption at 1 and 6 months in any group. -at 9 months, Group 1 and Group 2 showed 1/10 cases, while Group 3 showed none.
Goel et al. [25]	-In vivo -120 primary teeth from children aged 3–9	Group A: ZOE 4:1 ratio Group B: ZnO-Aloe vera 1:2 ratio Group C: ZnO-10%NaF Group D: Endoflas	No data	All four materials showed a general progressive radiographic healing, though differences between the groups were not statistically significant.	Root and filling material resorption were well matched in all groups, but zinc oxide–eugenol showed slower resorption and retained excess material longer than the others. Comparison of filling materials vs. root resorption 1. slower than root ZOE 96.70% ZnO-*Aloe vera* 11.10% ZnO-NaF 0%, Endoflas 0% 2. same as root ZOE 66.70%, ZnO-*Aloe vera* 77.80% ZnO-NaF 50%, Endoflas 100% 3. faster than the root ZOE 0%, ZnO-*Aloe vera* 11.1% ZnO-NaF 50% Endoflas 0%
Al-Ostwani et al. [26]	-In vivo -64 primary teeth from 39 children aged 3–9 -Divided into 4 group of 16 teeth each	Group 1: ZOE Group 2: Endoflas-chlorophenol-free Group 3: zinc oxide and propolis (ZOP pastes) Group 4: Metapex (calcium hydroxide with iodoform)	No data	Radiological success rate: After 6 months: ZOE -success 56.3% -suspicion 25% -failure 18.8% Endoflas-CF -success 81.3% -failure 18.8% ZOP -success 75% -suspicion 6.3% -failure 18.8% Metapex -success 75% -failure 25% After 12 months: ZOE -success 56.3% -suspicion 25% -failure 6.3% -extracted 12.5% Endoflas-CF -success 81.3% -failure 6.3% -extracted 12.5% ZOP -success 62.5% -suspicion 6.3% -failure 25% -extracted 6.3% Metapex -success 75% -failure 12.5% -extracted 12.5%	Clinical success rate after 6 months ZOE -success 93.8% -failure 6.3% Endoflas-CF -success 100% ZOP -success 100% Metapex -success 93.8% -failure 6.3% -extracted 0% After 6 months ZOE -success 87.5% -extracted 12.5% Endoflas-CF -success 87.5% -extracted 12.5% ZOP -success 93.8% -extracted 6.3% Metapex -success 87.5% -extracted 12.5% Comparison of filling materials vs. root resorption 1. slower than root ZOE 31.3% Endoflas CF 0% ZOP O% Metapex 0% 2. same as root ZOE 62.5% Endoflas CF 43.8% ZOP 37.5 Metapex 56.3% 3. faster than the root ZOE 6.3% Endoflas-CF 56.3% ZOP 37.5% Metapex 56.3%
Hegde et al. [52]	-In vitro -Research conducted on bacterial strains	Group 1: ZOE Group 2: ZnO + Ca(OH)_2_ + 10%NaF Group 3: ApexCal Group 4: Metapex Group 5: Endoflas Group 6: Vaseline (control group)	Zones of inhibition (mm): ZOE: Strong inhibition of *S. aureus* (19 ± 2.82), *S. epidermis* (19 ± 2.82), *B. subtilis* (20.5 ± 0.70), *P. aeruginosa* (18.5 ± 2.12), and *C. albicans* (26 ± 1.41); medium inhibition of other microorganisms. ZO + Ca(OH)_2_: No inhibition of *S. aureus*, *E. faecalis*, *P. aeruginosa*, *C. albicans*; weak inhibition of *S. epidermis* (6 ± 1.41); medium inhibition of others ZO + Ca(OH)_2_ + NaF: Medium inhibition of *S. epidermis* (12 ± 5.65), *S. mutans* (13.5 ± 0.70), *S. aureus* (7 ± 0), *B. subtilis* (7 ± 2.82); weak or no inhibition of others. ApexCal: Medium inhibition of *C. albicans* (12 ± 1.41), *S. epidermis* (9 ± 2.82), *B. subtilis* (9.5 ± 2.12); weak inhibition of *S. aureus* (3 ± 0), *S. mutans* 5 ± 2.82), *P. aeruginosa* (6 ± 1.41); no inhibition of *E. coli*, *E. faecalis*. Metapex: Mostly non-inhibitory; medium inhibition only against *B. subtilis* (10 ± 4.24). Endoflas: Strong inhibition of *C. albicans* (26 ± 1.41); medium inhibition of other microorganisms. Vaseline (control): No inhibition of any microorganisms.	No data	No data
Rojaramya et al. [34]	-In vivo -40 primary teeth from children aged 4–8	Group 1: zinc oxide-propolis mixture (ZOP) Group 2: ZOE (control)	No data	Radiological success rate: -After 6 months -ZOP 100% -ZOE 80% -After 12 months -ZOP 95% -ZOE 80% -After 24 months -ZOP 95% -ZnOE 70%	No data
Wasnik et al. [4]	-In vitro, -Study performed on bacterial strains	Tested materials: -Zinc Oxide Eugenol (ZOE) -Zinc oxide + *Morinda citrifolia* -Zinc oxide + *Aloe vera* -Zinc oxide + Neem -Control: Petroleum jelly (Vaseline)	Inhibition zone (mm): Against *S. aureus* (24 h): ZOE 20.66 mm (strong), ZnO + *M. citrifolia* 16.33 mm (medium), ZnO + *A. vera* 16.50 mm (medium), ZnO + Neem 16.33 mm (medium), Control 0 mm. Against *P. aeruginosa* (24 h): ZnO + *M. citrifolia* 28.50 mm (strong—most effective), ZnO + *A. vera* 25.83 mm (strong), ZnO + Neem 24.66 mm (strong), ZOE 24.33 mm (strong), Control 0 mm. Against *C. albicans* (24 h/48 h): ZOE 32.16/32.00 mm (strong), ZnO + *A. vera* 12.66/13.50 mm (medium), ZnO + Neem 11.66/12.50 mm (medium), ZnO + *M. citrifolia* 10.00/10.16 mm (weak), Control 0 mm.	No data	No data
Deepak et al. [37]	-In vitro -Research conducted on bacterial strain *Enterococcus faecalis*	Tested materials: -Group I: Zinc Oxide Eugenol paste (ZOE) -Group Ia: ZOE + 2.5% Triclosan -Group II: Endoflas -Group IIa: Endoflas FS + 2.5% Triclosan	Inhibition zone (mm): At 24 h: Group I 16.13 ± 1.60 mm, Group Ia 21.47 ± 1.77 mm, Group II 24.67 ± 2.44 mm, Group IIa 31.67 ± 3.16 mm (highest). At 6th Day (Day 7): Group I 14.4 ± 1.64 mm, Group Ia 16.53 ± 1.36 mm, Group II 20.93 ± 2.28 mm, Group IIa 27.60 ± 3.44 mm (highest). At 29th Day (Day 30): Group I 8.93 ± 2.60 mm (lowest) Group Ia 10.60 ± 3.23 mm, Group II 16.67 ± 2.02 mm, Group IIa 18.33 ± 2.02 mm (highest). Highest activity: 24 h post-mixing.	No data	No data
Kriplani et al. [36]	-In vitro -Microbiological samples collected from 20 primary molars with abscesses or sinus tracts in children aged 4–8 years, comprising facultative/aerobic Gram-positive (14 strains) and Gram-negative (4 strains) bacteria.	Tested materials: -*Aloe vera* + Sterile Water -Zinc Oxide Eugenol (ZOE) -ZOE + *Aloe vera* -Calcium Hydroxide + Sterile Water -Calcium Hydroxide + Sterile Water + *Aloe vera* -Metapex -Vaseline (control)	Inhibition Zone (mm): against all 18 Strains: *Aloe vera* + Water: range 10.83–27.66 mm (highest overall activity). ZOE + *Aloe vera*: range 11.5–25.66 mm (second highest). ZOE: range 9–20.66 mm. Ca(OH)_2_ + *Aloe vera*: range 0.66–21.16 mm. Ca(OH)_2_ + Water: range 0–12.16 mm (weakest among active materials). Metapex: range 0–10.33 mm (mostly ineffective). Vaseline: 0 mm (no inhibition—control). Ranking: Weak: 0.1–11.5 mm Medium: 11.5–19.7 mm Strong: >19.7 mm	No data	No data
Freire et al. [38]	-In vitro -5 samples of pastes based on zinc oxide	-Terpineol, -Cinnamaldehyde, -Terpineol + cinnamaldehyde -Chlorhexidine, -Chloramphenicol + tetracycline + zinc oxide + eugenol (control)	After 24 h, only the terpineol paste failed to inhibit *E. faecalis*, however after 72 h, all samples were effective. Both the MIC (Minimum Inhibitory Concentration) and MBC (Minimum Bactericidal Concentration) were 2000 μg/mL for terpineol and 500 μg/mL for cinnamaldehyde.	No data	After 24 h the terpineol paste turned out to be highly soluble, becoming completely dissolved within 48 h. After 144 h, the terpineol paste solubility was followed by the control paste. Other pastes did not show a significant difference in solubility.
Velasco-Loera et al. [50]	-In vitro -Microbial specimens collected from 21 deciduous teeth with at least one necrotic canal or sinus tract	-Group 1-Ultrapex -Group 2-metronidazole, ciprofloxacin, and minocycline	The modified paste showed a stronger antimicrobial effect, while Ultrapex exhibited minimal or no inhibition effect. However, *Clostridium ramosum* and *Candida albicans* turned out to be resistant to both examined materials.	No data	No data
Silva et al. [51]	-In vivo -40 teeth from 40 children aged 3–7.	-Calen + 1% CHX -Control—Calen	CFU Control 100% reduction Study group: Anaerobic bacteria—93.5% reduction Black-pigmented bacilli (BPB)—100% reduction Aerobic bacteria—96.5% reduction *Streptococci* (total)—92.4% reduction *Streptococcus mutans*—100% reduction	No data	CHX loses activity at high pH Ca(OH)_2_; possible reduced biocompatibility when combined
Antoniazzi et al. [2]	-In vitro -Research-tested, reference strains	G1—Control—Jodoform + Rifocort G2—Jodoform + Nebacetin G3—Jodoform + 2% CHX G4—Jodoform + Maxitrol	All tested pastes showed bacteriostatic activity against all microorganisms. G2, G3, and G4 performed similarly against *E. faecalis*, *E. coli*, *B. subtilis*, *S. oralis*, and *S. mutans*, while differences were observed for *S. aureus*, with G3 showing the strongest (10 ± 2.0 mm of inhibition zone) and G2 the weakest effect (5 ± 0.0 mm). All pastes were bactericidal against *E. coli*, *S. aureus*, *S. oralis*, and *S. mutans*. Only G3 and G4 were bactericidal against *E. faecalis*, and none showed bactericidal activity against *B. subtilis*.	No data	No data
Rivera-Albarrán et al. [5]	-In vitro -*Streptococcus mutans* (strains A, B, C, D) and *Enterococcus faecalis* (strains A, B, C, D, E) isolated from 34 primary teeth from children aged 6–10	-Guedes-Pinto modified (GPM) past -CTZ (chloramphenicol, tetracycline, zinc oxide eugenol) paste -Chlorhexidine-positive control -Distilled water-negative control	Inhibition zones varied among *S. mutans* and *E. faecalis* strains according to their antibiotic profiles. CTZ paste produced larger inhibition zones than GPM in most cases, though similar effects were observed for *S. mutans* C and *E. faecalis* D and E, and GPM was more effective against *S. mutans* D. Chlorhexidine showed consistently large inhibition zones, while distilled water produced minimal inhibition.	No data	No data
Jahan et al. [53]	-In vivo -90 primary teeth from 90 children aged 4–9.	-Group A Control—Zinc Oxide Eugenol (ZOE) -Group B ZOE + Calcium Hydroxide + Iodoform	No data	Radiographic outcomes: After 3 months: A: 39 teeth (43.3%)—reduction in radiolucency; 6 teeth (6.7%)—no improvement B: 45 teeth (100%)—reduction in radiolucency; 0 teeth (0%)—no improvement After 6 months: A: 39 teeth (43.3%)—reduction in radiolucency; 6 teeth (6.7%)—no improvement B: 45 teeth (100%)—reduction in radiolucency; 0 teeth (0%)—no improvement	Pain status Before 100% After 3 months: A: 88.9% B: 0% After 6 months: no pain in both groups Tenderness on percussion Before: 100% After 3 months: A: 77.8% B: 0% After 6 months: none Gingival swelling: Before: A: 23.3% B: 22.2% After 3 months: A: 1% B: 0% After 6 months no swelling in both groups

In summary, the in vitro analysis indicates that zinc oxide–eugenol remains the most commonly used reference material regarding antimicrobial efficacy [4,36,52]. Modifications of resorbable pastes with plant extracts, antibiotics, or triclosan can significantly enhance biological activity, although the effect depends on time and strain [2,4,5,37,50]. Physicochemical properties, particularly solubility, represent a key differentiating factor among materials and may affect their potential clinical applicability [38,48].

#### 3.3.2. In Vivo Studies—Detailed Outcomes

Qualified in vivo clinical studies focused on the evaluation of the clinical effectiveness of modified, resorbable materials used for root canal filling in pulpectomy of primary teeth [1,24,25,26,27,34,51,53]. Clinical outcomes were primarily assessed based on the presence or absence of pain, swelling, tenderness on percussion, sinus tract formation, pathological mobility, and signs of infection, while radiographic outcomes focused on changes in interradicular or periapical radiolucency and bone density.

The studies differed in terms of sample size, age of participants, and follow-up period. Despite these differences, most of the modified tested materials, (with the addition of propolis, *Aloe vera*, antibiotics, or ozonated olive oil) [1,25,26,27,34,51], demonstrated high clinical and radiographic effectiveness. A common trend was the gradual resolution of clinical symptoms, such as pain and tenderness, over several to twelve months of observation, regardless of the type of material used.

Radiographic analysis showed that modified formulations often accelerated the reduction in interradicular radiolucency and supported better bone density gain compared to conventional materials. In some studies, pastes containing propolis, ozonated oil, or antibiotics achieved higher radiographic effectiveness, although the differences between groups were not always statistically significant [24,27,34,50].

Several studies also evaluated the degree of material resorption. Appropriate resorption in primary teeth is clinically relevant to avoid interference with physiological root resorption and eruption of permanent teeth. Reported resorption indices varied between studies: some authors found no significant differences between conventional and modified materials [49]. Whereas others observed that ZOE tended to resorb more slowly than alternative formulations, potentially leading to material remnants in periapical tissues [53]. Such differences may influence long-term radiographic appearance and treatment interpretation.

It should be noted, however, that modifications do not always improve material properties; certain additives may even reduce effectiveness. For example, CHX added to calcium hydroxide at high pH loses its antimicrobial properties [51].

In light of the presented data, material modifications can enhance both clinical and radiographic effectiveness, although the effect depends on the type of additive, tooth type, follow-up duration, and the microbial resistance profile.

### 3.4. Quality Assessment of Included Studies

Among in vivo studies, for all the five questions, 3 papers received a positive answer to five of them [24,26,36], 4 papers received a positive answer to four of them [1,27,51,53], while 1 article received a positive answer to three of them [25]. As for the in vitro study only the applicable criteria were applied, all 10 studies received 3 positive answers to all 3 relevant criteria for this kind of study [2,4,5,36,37,38,48,49,50,52] (see Table 3).

Overall, MMAT scores should be interpreted with caution, particularly for in vitro studies, as only a subset of criteria was applicable. While most in vitro studies fulfilled all relevant MMAT items, this primarily reflects adequate reporting of experimental procedures rather than low risk of bias in a clinical sense. Among in vivo studies, lower scores were mainly driven by limitations related to a lack of blinding and incomplete reporting of randomization procedures.

## 4. Discussion

The findings of this systematic review indicate that modifications of resorbable root canal filling materials for primary teeth can significantly influence their microbiological performance. Such modifications include the incorporation of antimicrobial additives (e.g., triclosan, phytoextracts, or antibiotics) or alterations to the base matrix of iodoform- and iodide-containing pastes, which can also affect their clinical behavior in many cases [1,2,5,24,25,26,27,34,36,37,38,48,49,50,53]. Several in vitro studies confirmed enhanced antibacterial effects following the incorporation of triclosan [37], cinnamaldehyde and terpineol–cinnamaldehyde combinations [38], or herbal extracts in zinc oxide–based formulations [4]. Similarly, modifications of calcium hydroxide–iodoform systems have demonstrated improved antimicrobial properties and favorable physicochemical characteristics [26,48,49,50]. Recent studies also suggest that bioactive additives can enhance the ability of root canal materials to inhibit pathogens commonly associated with endodontic infections, including *Enterococcus faecalis* [39,54,55,56]. Clinical investigations further support the potential benefits of modified formulations. Propolis-enriched zinc oxide pastes, phytotherapeutic combinations, and mixed calcium–iodine preparations have achieved high clinical and radiographic success rates, in some cases outperforming conventional ZOE or performing comparably to materials such as Vitapex [5,24,25,26,34,36,37,53]. Collectively, the evidence shows a trend toward enhancing the bioactivity and antimicrobial properties of obturation materials for primary teeth. This highlights the need for formulations that effectively address persistent microbial challenges while ensuring favorable clinical outcomes.

In several cases within the studies considered, there was a noticeable discrepancy between the antibacterial efficacy demonstrated in laboratory conditions and clinical efficacy. For example, while many zinc oxide-based preparations showed strong inhibition of Enterococcus faecalis and Candida albicans in vitro [2,4,5,37,38,49], corresponding in vivo studies did not consistently show proportionally better clinical results compared to conventional zinc oxide and eugenol [24,25,53]. Similarly, antibiotic-enriched pastes showed strong antibacterial activity under laboratory conditions [50], but their clinical superiority was variable and limited by concerns about the potential development of bacterial resistance [5], which could not be fully assessed under controlled in vitro conditions. This gap likely reflects the complexity of the intraoral environment—including local pH changes, inflammatory responses, tissue barriers, and dynamic host–pathogen interactions—factors that cannot be fully replicated under standard laboratory conditions. Therefore, strong in vitro antibacterial activity alone should not be interpreted as a reliable indicator of clinical efficacy. This discrepancy highlights the critical importance of well-designed in vivo studies with extended follow-up periods to confirm laboratory findings and determine the true clinical significance of material modifications in primary tooth endodontics.

Interpreting these findings within the broader scientific context shows clear alignment with current trends in endodontic material development. Numerous authors underline the limitations of traditional materials such as ZOE and iodoform-based pastes, including insufficient antimicrobial activity, unpredictable resorption rates, and undesirable reactions within periapical tissues [25,48,50,57]. The results of the present review corroborate these concerns and further suggest that contemporary formulations—such as sodium iodide–based pastes modified with lanolin—can achieve ISO-compliant physicochemical standards while exhibiting improved solubility profiles and enhanced material stability [48]. Emerging evidence also indicates that bioactive additives may strengthen mechanical and adhesive properties, potentially improving the long-term performance of root canal fillings in primary teeth [50,58]. From a clinical perspective, these advancements are particularly relevant given the anatomical complexity of primary root canals and the frequent challenge of incomplete instrumentation [59,60,61,62,63]. High antimicrobial efficacy of filling materials is therefore essential [1,37,38,51]. Clinical and radiographic studies—including those by Antoniazzi, Kriplani, Jahan, and Singh—demonstrated that modified materials can maintain favorable outcomes even in cases presenting with pronounced periapical pathology [1,2,4,5,24,25,36,53]. Recent meta-analyses further support the notion that obturation materials with enhanced bioactivity and broader antimicrobial spectra may increase the success rates of pulpectomy procedures, especially in teeth affected by sinus tracts or advanced inflammatory lesions [39]. Additionally, Thakur et al. argue that modern bioactive pastes may offer greater clinical predictability compared with conventional calcium–iodine formulations [64].

Despite the promising results, several important limitations of the available studies must be acknowledged. First, there is substantial methodological heterogeneity, particularly among in vitro experiments. The studies employed different microbial strains, variable inoculum preparation, and non-standardized protocols for determining MIC and MBC values, solubility, and compliance with ISO requirements, which hampers direct comparison of outcomes across investigations [2,25,26,37,38,48,49,50,51]. Second, relatively few studies have examined the long-term clinical performance of modified materials; many rely on short follow-up periods or retrospective assessments, limiting the ability to draw robust conclusions regarding durability and long-term healing patterns [1,5,24,25,34,36,53]. Another critical limitation is the lack of evaluation of potential effects on physiological root resorption and the development of the permanent successor tooth—an essential consideration for ensuring the safety of pulpectomy procedures in primary dentition [5,34,48,50,65]. Moreover, no comprehensive studies have systematically compared the resorption behavior of materials with different base matrices. These include iodide-based pastes, iodoform derivatives, modified ZOE formulations, and phytotherapeutic combinations. This lack of data limits our understanding of how material composition affects degradation kinetics and interactions with periapical tissues. While included studies achieved high MMAT quality scores, important limitations affect robustness. Many in vitro studies used relatively small numbers of bacterial isolates, limiting generalizability across diverse endodontic pathogens. Heterogeneous study designs and outcome measures precluded direct comparisons. Although in vivo studies reported adequate sample sizes, follow-up periods were often limited to ≤12 months, insufficient for assessing long-term material performance. These factors—limited in vitro sampling, methodological heterogeneity, short clinical follow-ups—indicate that, despite promising evidence for modified materials, definitive clinical superiority claims require larger, longer-term standardized trials.

This systematic review has several limitations that should be considered when interpreting the findings. First, substantial methodological heterogeneity was observed among the included studies, particularly with respect to study design, sample characteristics, material formulations, and outcome assessment methods. This heterogeneity precluded quantitative synthesis and limited direct comparison across studies. It also complicates clinical decision, because of the effectiveness of materials observed under experimental conditions does not always translate to clinical outcomes. In vitro studies have demonstrated that modified zinc oxide-based pastes, including those with added triclosan or plant extracts, strongly inhibit the growth of microorganisms such as *Enterococcus faecalis* and [2,4,5,36,37]. However, in vivo studies demonstrated clinical efficacy similar to conventional materials such as ZOE [24,25]. For pastes enriched with natural components, such as propolis or *Aloe vera*, accelerated bone healing and a reduction in inflammatory symptoms were observed [25,27,34]. These findings point out the gap between in vitro potential and actual clinical outcomes. The lack of studies assessing the same additives both in vitro and in vivo further complicates the interpretation of results. Clinicians should therefore carefully evaluate laboratory findings and short-term clinical studies, taking into account factors such as patient age, tooth type, and local characteristics of the infection. The most promising approach appears to be the modification of zinc oxide with plant extracts. ZOE has been used for several decades in pediatric dentistry with a high success rate. Its main disadvantage is the release of free eugenol, which at high concentrations exhibits cytotoxic effects on fibroblasts and osteoblast-like cells [27]. This limitation has driven researchers to seek alternatives to eugenol and to develop chemical compounds with broader and stronger antibacterial activity. Naturally derived materials, such as propolis [34], *Aloe vera* [25], and olive oil—both ozonated and non-ozonated [27], have demonstrated satisfactory clinical and radiographic outcomes in in vivo studies. Bone regeneration and a reduction or complete resolution of inflammatory symptoms, including spontaneous pain, pain on percussion, and abnormal tooth mobility, were observed even in follow-up periods of up to 24 months.

In addition, the search strategy was deliberately designed to be sensitive to antimicrobial modifications, which may have resulted in the underrepresentation of studies focusing primarily on physicochemical or clinical outcomes without explicit antimicrobial terminology.

Second, a considerable proportion of the included evidence was derived from in vitro investigations. Although such studies provide valuable insights into antimicrobial activity and physicochemical properties of modified materials, their findings cannot be directly extrapolated to clinical performance. Even among in vivo studies, variability in follow-up duration, outcome definitions, and radiographic assessment criteria may have influenced the reported success rates.

Third, the lack of standardized outcome measures across studies represents an important limitation. Differences in microbiological testing protocols, clinical success criteria, and radiographic evaluation methods may affect the generalizability of the results. In addition, some studies included relatively small sample sizes or short follow-up periods, which may limit the strength of clinical conclusions.

Finally, despite adherence to PRISMA 2020 guidelines [42], the possibility of publication bias cannot be excluded, as studies reporting favorable outcomes of material modifications may be more likely to be published. These limitations highlight the need for well-designed, long-term randomized clinical trials with standardized methodologies to better evaluate the clinical relevance of modified resorbable root canal filling materials for primary teeth.

Future research should focus on developing antimicrobial strategies without the use of antibiotics to address the problem of bacterial resistance associated with antibiotic-containing preparations, including systematic research into natural bioactive compounds, metal-based nanoparticles, and antimicrobial peptides [34,50,66]. Additionally, long-term clinical studies extending until physiological exfoliation with standardized follow-up periods of at least 24–36 months are essential to assess material resorption kinetics relative to natural root resorption, clinical and radiographic outcomes, and effects on permanent successor tooth development [39,64]. To increase the comparability of studies and reduce methodological differences, it is necessary to use standard in vitro test protocols compliant with ISO 6876 and using consistent bacterial strains, preparation methods, and test methodologies [36,48,49,50]. Finally, mechanistic investigations examining material-tissue interactions, biofilm formation dynamics, and the influence of local pH and inflammatory mediators on antimicrobial efficacy and resorption behavior would clarify the translational gap between laboratory findings and clinical performance, ultimately supporting more predictable clinical translation of modified materials [67,68,69].

## 5. Conclusions

This systematic review indicates that various modifications of resorbable root canal filling materials demonstrate enhanced antimicrobial activity and favorable physicochemical properties under laboratory conditions. In vitro evidence suggests that the incorporation of bioactive additives, alternative base components, or modified formulations may improve antimicrobial performance compared with conventional materials.

However, the available in vivo evidence remains limited and heterogeneous. Although several clinical studies report satisfactory clinical and radiographic outcomes for selected modified materials, these findings should be interpreted with caution due to variability in study design, outcome definitions, and follow-up duration. Importantly, the predominance of laboratory-based evidence limits the extent to which clinical effectiveness can be conclusively established.

Overall, while material modifications show promising potential, current evidence does not allow definitive conclusions regarding their clinical superiority over conventional formulations. Well-designed, long-term randomized clinical trials with standardized methodologies are required to confirm whether laboratory-observed advantages translate into consistent clinical benefits in primary teeth.

## Figures and Tables

**Table 1:** General characteristics of included studies.

Study	Aim of the Study	Material and Methods	Conclusions
Alashbal et al. [49]	In vitro evaluation of the antimicrobial potential of a zinc oxide–eucalyptus oil paste compared with conventional zinc oxide–eugenol paste and Metapex for root canal obturation in primary teeth.	The in vitro study was conducted on *Streptococcus* spp. and *Candida albicans* strains isolated from 21 necrotic primary teeth of children aged 3–8 years, with or without periapical lesions. The tested materials were placed on agar plates inoculated with the microorganisms, and the diameter of the inhibition zones was measured after overnight incubation at 37 °C.	The experimental paste zinc oxide–eucalyptus oil paste, exhibited an antimicrobial effect comparable to that of zinc oxide–eugenol. Metapex showed the lowest antimicrobial activity against the tested microorganisms.
Park et al. [48]	Evaluation of the physicochemical properties of an experimental root canal pastes containing sodium iodide (instead of iodoform) with and without lanolin, in comparison with Vitapex.	Based on evaluations of pastes with different compositions and ingredients, two formulations were selected for study: L0 group, which exhibited the lowest solubility and contained calcium hydroxide, sodium iodide, and lanolin, and L5 group, which had the same composition without lanolin, compared to Vitapex as the control. Physicochemical properties were evaluated according to ISO 6876:2012, including solubility, flow, film thickness, radiopacity, injectability, viscosity, pH changes, ion release, root canal filling ability, removability of the paste from the canal, and antibacterial activity against *Enterococcus faecalis*.	The experimental sodium iodide–based pastes may serve as an alternative for filling the root canals of primary teeth compared to conventional materials such as Vitapex. However, further in vivo biocompatibility studies are required before clinical application.
EL-Desouky et al. [27]	In vivo evaluation of the clinical and radiographic efficacy of two experimental zinc oxide pastes: with ozonated olive oil and with non-ozonated olive oil for root canal obturation in primary teeth.	In a randomized study, 90 s primary molars of children aged 4–8 years were divided into three groups and obturated with the following materials: zinc oxide–ozonated olive oil, zinc oxide–olive oil, and zinc oxide–eugenol (control). Clinical and radiographic assessments were conducted at 3, 6, and 12 months.	The mixtures of zinc oxide with ozonated and non-ozonated olive oil are more effective as root canal filling materials in primary teeth than conventional zinc oxide with eugenol.
Pinky et al. [1]	In vivo evaluation of the clinical and radiographic efficacy of two antibiotic mixtures in the infected primary teeth treatment.	In the study, 40 primary molars of children aged 4–10 years were obturated with a paste containing ciprofloxacin, metronidazole, and minocycline, or with a paste containing ciprofloxacin, ornidazole, and minocycline. Clinical evaluations were performed at 15 and 30 days, followed by both clinical and radiographic assessments at 3, 6, and 12 months.	The paste containing ornidazole instead of metronidazole demonstrated better clinical efficacy but requires further studies with longer follow-up periods until tooth exfoliation.
Singh et al. [24]	In vivo assessment of the clinical and radiographic efficacy of zinc oxide–propolis paste compared with zinc oxide–eugenol and Endoflas in deciduous teeth pulpectomy.	In the study, 60 primary molars from children aged 4–9 years after pulpectomy were obturated with zinc oxide–eugenol, zinc oxide–propolis and Endoflas. Clinical and radiographic results were evaluated at 3, 6, and 9 months.	The combination of zinc oxide with and propolis and Endoflas are more effective materials than traditional zinc oxide with eugenol.
Goel et al. [25]	To compare four different zinc oxide integrated root canal obturating materials.	The effects of treatment of 120 primary molars with irreversible pulpitis filled with zinc oxide eugenol, zinc oxide powder with 10% sodium fluoride, zinc oxide powder with *Aloe vera* and Endoflas. Single sitting pulpectomy was carried out and restored with a preformed crown in next visit.	Sodium fluoride and aloe vera can serve as effective, low-cost alternatives to root canal obturating materials for primary teeth.
Al-Ostwani et al. [26]	To assess the effectiveness of pulpectomy in nonvital primary molars using 4 different root canal filling materials: zinc oxide and propolis, Endoflas-chlorophenol-free, Metapex paste, and zinc oxide and eugenol	The study involved 64 nonvital primary molars randomly assigned to four groups based on the root canal filling material. Pulpectomy was completed in a single session using 5.25% sodium hypochlorite for irrigation and restored with stainless-steel crowns	Zinc oxide-propolis paste showed strong potential due to its natural antibacterial properties, while zinc oxide-eugenol performed comparably to the other material
Hegde et al. [52]	To compare the antimicrobial effectiveness of 6 commonly used root canal filling materials for primary teeth against typical infection-causing microorganisms.	The in vitro experiment tested the antimicrobial effects of 6 root canal filling materials (zinc oxide and eugenol paste, a mixture of zinc oxide powder and calcium hydroxide paste in distilled water, a mixture of zinc oxide powder and calcium hydroxide paste in 10% sodium fluoride, calcium hydroxide paste, calcium hydroxide with iodoform, and a mixture of iod-oform, calcium hydroxide and zinc oxide) against 8 microbial strains using agar diffusion.	The tested filling materials showed different levels of antimicrobial activity, with zinc oxide-based materials working better against the microorganisms than those without zinc oxide.
Rojaramya et al. [34]	To evaluate and compare how well a zinc oxide-propolis mixture works as a root canal filling material in non-vital primary molars, compared to traditional zinc oxide eugenol (ZOE).	Forty primary molars needing pulpectomies were split into two groups: one received a zinc oxide-propolis mixture, and the other received ZOE. All teeth were restored with stainless steel crowns, and children were checked at 6, 12, and 24 months. A Chi-square test was used to analyze the results.	Zinc oxide-propolis mixture showed good clinical and radiographic success after 24 months, suggesting it could be a suitable alternative root canal filling material for primary teeth.
Wasnik et al. [4]	The antimicrobial efficacy of zinc oxide eugenol (ZOE) and zinc oxide mixed with *Morinda citrifolia*, *Aloe vera*, or neem extracts.	MIC determined via broth dilution. Test materials: zinc oxide mixed with MIC of extracts (*M. citrifolia* 6.25%, *A. vera* 6.25–25%, neem 6.25–12.5%) compared to ZOE and vaseline (control). Agar diffusion was performed using 3 mm wells. Inhibition zones measured after incubation at 37 °C: 24 h for S. aureus and *P. aeruginosa*; 24/48 h for *C. albicans.*	ZOE proved most effective against *S. aureus* and *C. albicans*, while zinc oxide combined with *M. citrifolia* outperformed ZOE against *P. aeruginosa*. The herbal extract combinations mixed with zinc oxide show potential as alternative root canal filling materials for primary teeth.
Deepak et al. [37]	To compare the antibacterial activity of zinc oxide eugenol (ZOE) and Endoflas FS with/without 2.5% triclosan incorporation against *Enterococcus faecalis*.	Four groups (*n* = 15): ZOE, ZOE + 2.5% triclosan, Endoflas FS, Endoflas FS + 2.5% triclosan. Double-layer agar well diffusion tested against *E. faecalis* using five 10 × 4 mm wells per plate, incubated at 37 °C for 24 h. Testing at 24 h, day 6, and day 29 with fresh plates; specimens rinsed and stored in sterile water between tests.	Incorporating 2.5% triclosan enhanced the antimicrobial activity of ZOE and Endoflas FS against *E. faecalis*. Endoflas FS with triclosan demonstrated superior antibacterial efficacy. All materials maintained sustained antimicrobial activity through day 29, effectiveness gradually decreased. Triclosan addition to root canal filling materials improves the elimination of residual microflora.
Kriplani et al. [36]	To calculate the MIC of *Aloe vera* and evaluate the antimicrobial efficacy of six root canal filling materials (*Aloe vera* + water, ZOE, ZOE + *Aloe vera*, calcium hydroxide + water, calcium hydroxide + water + *Aloe vera*, Metapex) and vaseline control against bacteria isolated from infected primary teeth.	*Aloe vera* was processed and sterilized; MIC = 400 mg/mL, MBC = 500 mg/mL. Eighteen bacterial strains (14 Gram-positive, 4 Gram-negative) were isolated from 20 infected deciduous molars in children aged 4–8 years. Inhibition zones were measured at 16–24 h and categorized as: None (0 mm), Weak (0.1–11.5 mm), Medium (11.5–19.7 mm), or Strong (>19.7 mm).	*Aloe vera* + water showed the strongest antimicrobial activity, followed by ZOE + *Aloe vera*, calcium hydroxide + Aloe vera, ZOE, calcium hydroxide, and Metapex; vaseline was inactive. *Aloe vera* is a potential root canal filling material for primary teeth and significantly enhances ZOE and calcium hydroxide’s antimicrobial effectiveness against bacteria from infected primary teeth.
Freire et al. [38]	Evaluation of the solubility and antibacterial effect of experimental root canal pastes with terpineol and cinnamaldehyde compared to conventional paste in primary teeth.	Five zinc oxide-based pastes were prepared (terpineol, cinnamaldehyde, terpineol + cinnamaldehyde, chlorhexidine, chloramphenicol+ tetracycline + zinc oxide + eugenol). Solubility was measured with a spectrophotometer after 48 h and 144 h immersion of the specimens in distilled water at 37 °C. Antibacterial activity was tested by the direct contact method using *E. faecalis* after 24 h and 72 h.	Experimental pastes containing cinnamaldehyde or terpineol + cinnamaldehyde showed CTZ-comparable antibacterial activity and lower solubility.
Velasco-Loera et al. [50]	Evaluation of antibacterial effect of a modified paste (zinc oxide with (metronidazole, ciprofloxacin and minocycline)) compared to iodoform paste.	Control and experimental pastes were sandwiched between two filter paper disks. The disk-diffusion tests were performed in an anaerobic chamber using 21 microbial samples from necrotic pulp canals. Disks were incubated at 35 °C for 48 h.	The experimental paste (zinc oxide with (metronidazole, ciprofloxacin and minocycline)) exhibited a superior antibacterial effect compared to the iodoform paste.
Silva et al. [51]	In vivo evaluation of the antibacterial effect of calcium hydroxide (CH) with or without chlorhexidine (CHX) in primary teeth with periapical inflammation.	In a randomized study, 40 root canals in children aged 3–7 years were filled with CH or CH + CHX paste for 30 days, and the number and elimination of microorganisms in microbiological samples taken before and after treatment were compared.	The addition of chlorhexidine to calcium hydroxide does not provide additional antibacterial benefits in root canal treatment of primary teeth with periapical inflammation.
Antoniazzi et al. [2]	Assessment of the antibacterial potential of filling pastes for primary teeth in which the withdrawn ingredient Rifocort was replaced with other preparations with known antimicrobial activity.	In vitro tests were performed using the diffusion method, comparing four paste combinations (with Rifocort, Nebacetin, 2% chlorhexidine and Maxitrol) against six strains of aerobic and facultatively anaerobic bacteria.	All tested pastes have antibacterial potential suitable for use in endodontic treatment of primary teeth, but require further evaluation for biocompatibility before clinical use.
Rivera-Albarrán et al. [5]	Assessment of the influence of bacterial resistance on the effectiveness of antibiotic filling pastes used in root canal treatment of primary teeth.	An in vitro study was conducted with Streptococcus mutans and Enterococcus faecalis isolated from the root canals of necrotic teeth of 34 children, identifying their resistance profile to tetracycline, chloramphenicol and rifampicin, and then testing the activity of CTZ and Guedes-Pinto modified (GPM) toothpastes against sensitive and resistant strains.	Bacterial resistance to antibiotics reduces the effectiveness of antibiotic endodontic pastes, which undermines the validity of simplified root canal treatment techniques based on such preparations and indicates the need to develop alternative methods without the use of antibiotics.
Jahan et al. [53]	Evaluation of the therapeutic efficacy of a mixture of zinc oxide with eugenol, calcium hydroxide and iodoform in comparison with traditional zinc oxide with eugenol in root canal treatment of primary teeth.	In an experimental study, 90 children aged 4–9 years underwent pulpectomy using ZOE (control) and ZOE with Ca(OH)_2_ and iodoform in two comparative groups, respectively; clinical and radiological results were assessed after 3 and 6 months.	The combination of zinc oxide with eugenol, calcium hydroxide and iodoform is a more effective and safe filling material in pulpectomy of primary teeth than the classic zinc oxide with eugenol.

**Table 3:** Quality assessment of Included Studies.

Quantitative Randomized Controlled Trials	Methodological Quality Criteria				
Authors	1. Is randomization appropriately performed?	2. Are the groups compared at the baseline?	3. Are there complete outcome data?	4. Are outcome assessors blinded to the intervention provided?	5. Did the participants adhere to the assigned intervention?
Al-Ostwani et al. [26]	Yes	Yes	Yes	Yes	Yes
Pinky et al. [1]	Yes	Yes	Yes	No	Yes
Alshabal et al. [49]	Yes	Not applicable	Yes	Yes	Not applicable
El-Desouky et al. [27]	Yes	Yes	No	Yes	Yes
Goel et al. [25]	Yes	Yes	No	No	Yes
Jahan et al. [53]	Yes	Yes	Yes	No	Yes
Rojaramya et al. [34]	Yes	Yes	Yes	Yes	Yes
Silva et al. [51]	Yes	Yes	No	Yes	Yes
Singh et al. [24]	Yes	Yes	Yes	Yes	Yes
Quantitative descriptive	Methodological quality criteria				
Authors	1. Is the sampling strategy relevant to address the research question?	2. Is the sample representative of the target population?	3. Are the measurements appropriate?	4. Is the risk of nonresponse bias low?	5. Is the statistical analysis appropriate to answer the research question?
Park et al. [48]	Yes	Not applicable	Yes	Not applicable	Yes
Hegde et al. [52]	Yes	Not applicable	Yes	Not applicable	Yes
Wasnik et al. [4]	Yes	Not applicable	Yes	Not applicable	Yes
Deepak et al. [37]	Yes	Not applicable	Yes	Not applicable	Yes
Kriplani et al. [36]	Yes	Not applicable	Yes	Not applicable	Yes
Freire et al. [38]	Yes	Not applicable	Yes	Not applicable	Yes
Velasco-Loera et al. [50]	Yes	Not applicable	Yes	Not applicable	Yes
Antoniazzi et al. [2]	Yes	Not applicable	Yes	Not applicable	Yes
Rivera-Albarrán et al. [5]	Yes	Not applicable	Yes	Not applicable	Yes

## Data Availability

The original contributions presented in this study are included in the article/Supplementary Material. Further inquiries can be directed to the corresponding authors.

## Supplementary Materials

The following supporting information can be downloaded at: https://www.mdpi.com/article/10.3390/ma19050950/s1: PRISMA 2020 Checklist.

## Abbreviations

The following abbreviations are used in this manuscript:

WoS	Web of Science
NiTi	Nickel-Titanum
NaOCl	sodium hypochlorite
CHX	Chlorheksidine
EDTA	ethylenediaminetetraacetic
NRSs	Non-randomized controlled clinical trials
RCTs	Randomized controlled clinical trials
CTZ	Chloramphenicol, tetracycline, zinc oxide
ZOE	Zinc oxide-eugenol
MIC	Minimal Inhibitory Concentration
MBC	Minimum Bactericidal Concentration
